# Supplementary figures and images for: Preclinical evaluation of a protracted GLP-1/glucagon receptor co-agonist: Translational difficulties and pitfalls
Source: PLoS One. 2022 Mar 4;17(3):e0264974. doi: 10.1371/journal.pone.0264974 (PMC8896685; doi:10.1371/journal.pone.0264974)

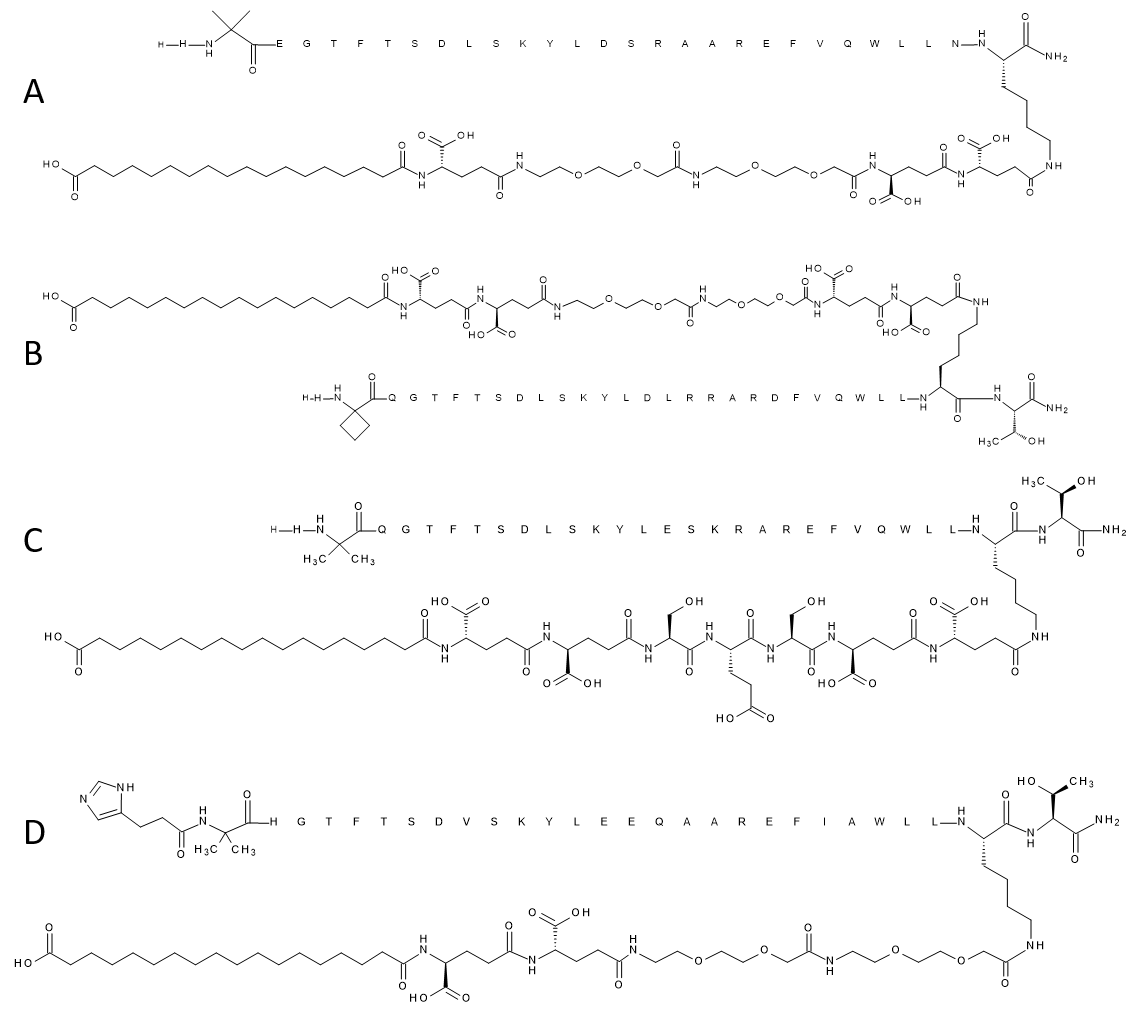

Supplement: S1 Fig — Structures of the A) GLP-1 reference compound B) co-agonist NN1151, C) co-agonist NN1177 and D) co-agonist NN1359. (TIF) [file pone.0264974.s001.tif]

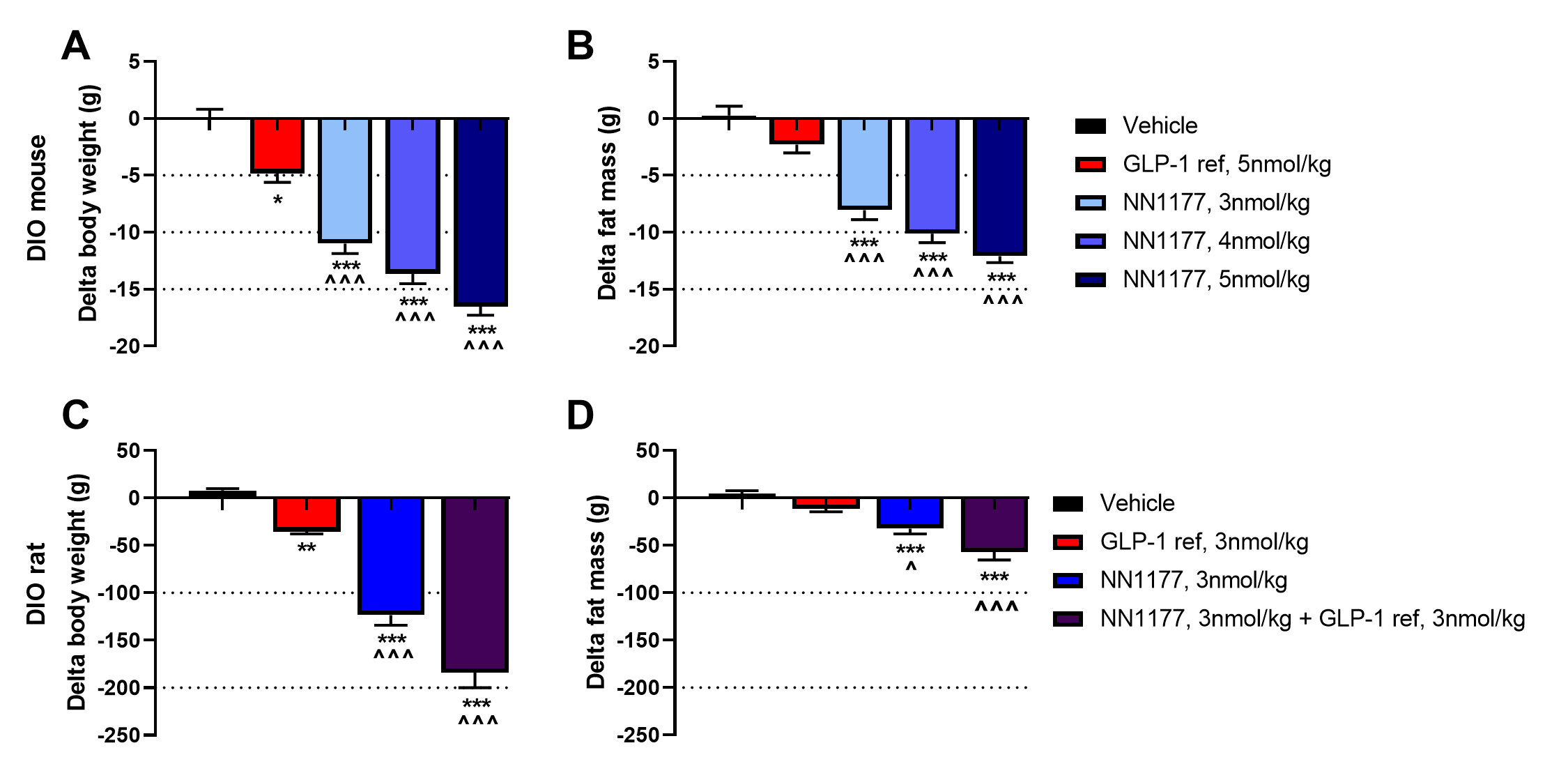

Supplement: S2 Fig — Delta body weight (A, C) and delta body fat (B, D) of DIO mice (A, B) and DIO rats (C, D) treated daily s.c. for 3–5 weeks with vehicle, GLP-1 reference, NN1177 or a combination of NN1177 and GLP-1 reference (n = 8–20). Percentage indicates the amount of weight loss that can be explained by loss of fat mass. *p<0.05, **p<0.01, ***p<0.001 vs vehicle; ^p<0.05, ^^^p<0.001 vs the GLP-1 reference compound. (TIF) [file pone.0264974.s002.tif]

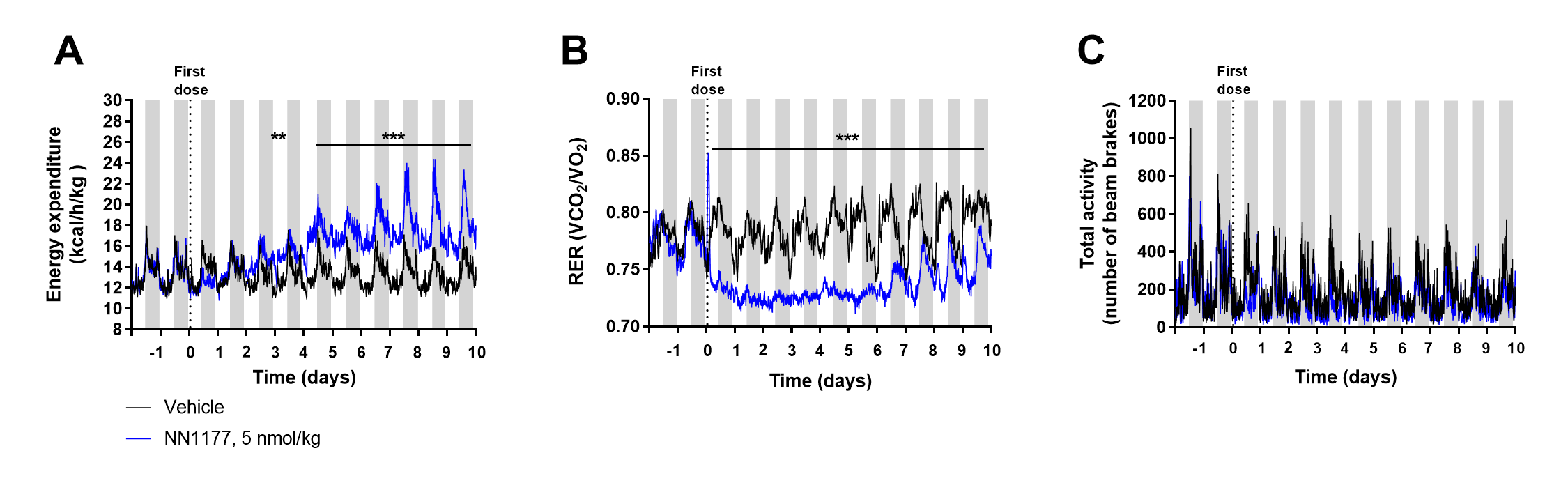

Supplement: S3 Fig — Energy expenditure per body weight (A), respiratory exchange ratio (RER) (B) and activity (C) in DIO mice treated once daily s.c. with vehicle or NN1177 (n = 8). **p<0.01; *** p<0.001 vs vehicle, two-way ANOVA based on mean light/dark values (average values shown). (TIF) [file pone.0264974.s003.tif]
