# Supplementary material for: Preclinical evaluation of a protracted GLP-1/glucagon receptor co-agonist: Translational difficulties and pitfalls
Source: PLoS One. 2022 Mar 4;17(3):e0264974. doi: 10.1371/journal.pone.0264974 (PMC8896685; doi:10.1371/journal.pone.0264974)
Supplement: S1 Table — (DOCX) [file pone.0264974.s004.docx]

S1 Table. Receptor potency at rat GLP-1 and glucagon receptors

|  | GLP-1R  EC_50_, pM  (95% CI)  0% plasma | GCGR  EC_50_, pM  (95% CI)  0% plasma | Normalized Ratio^a^ |
| --- | --- | --- | --- |
| NN1177 | 0.61 (0.45-0.81) | 1.83 (1.16-2.89) | 1:3 |
| GLP-1 reference | 2.99 (1.51-5.92) | 35300 (18900-66000) | n/a |
| GLP-1 | 1.35 (1.07-1.70) | n/a |  |
| Glucagon | 156 (123-197) | 1.49 (1.42-1.57) | n/a |

n/a, not applicable

**^a^** Ratio was calculated on EC_50_ levels after normalization to the endogenous ligand on each receptor, to account for slight differences in the assay systems and reported as the ratio of GLP-1R:GCGR EC_50_ values.
