# Supplementary material for: Preclinical evaluation of a protracted GLP-1/glucagon receptor co-agonist: Translational difficulties and pitfalls
Source: PLoS One. 2022 Mar 4;17(3):e0264974. doi: 10.1371/journal.pone.0264974 (PMC8896685; doi:10.1371/journal.pone.0264974)
Supplement: S2 Table — (DOCX) [file pone.0264974.s005.docx]

**S2 Table: Tissue distribution of glucagon receptors in rat and mouse**

| **GCGR** | **Study** | **Adrenal gland** | **Adipose** | **Bladder** | **Brain cereb.** | **Brain cortex** | **Heart** | **GI tract** | **Kidney** | **Liver** | **Lung** | **Muscle** | **Ovary** | **Pancreas (Islets)** | **Pituitary** | **Spleen** | **Stomach** | **Testes** | **Thymus** | **Thyroid** |
| --- | --- | --- | --- | --- | --- | --- | --- | --- | --- | --- | --- | --- | --- | --- | --- | --- | --- | --- | --- | --- |
| **Rat** | Söllner *et al*.[1] | NA | NA | NA | - | | + | - | + | + | NA | - | NA | - | NA | NA | +/- | NA | + | NA |
| **Rat** | Roller *et al.*[2] | NA | NA | NA | - | | NA | NA | NA | + | NA | - | NA | NA | NA | NA | NA | - | NA | NA |
| **Rat** | Naqvi *et al*.[3] | - | +/- | - | - | | + | NA | NA | + | - | +/- | NA | NA | - | + | NA | - | NA | + |
| **Rat** | Dunphy *et al*.[4] | + | NA | NA | - | +/- | NA | +/- | + | + | +/- | NA | NA | +/- | NA | + | + | - | + | NA |
| **Rat** | Hansen *et al*.[5] | +/- | + | NA | - | - | + | + | + | + | - | +/- | +/- | +/- | NA | + | +/- | - | +/- | +/- |
| **Mouse** | Söllner *et al.*[1] | NA | NA | NA | - | | - | - | + | + | NA | - | NA | - | NA | NA | - | NA | - | NA |
| **Mouse** | Roller *et al*.[2] | NA | NA | NA | - | | NA | NA | NA | + | NA | - | NA | NA | NA | NA | NA | - | NA | NA |
| **Mouse** | Naqvi *et al.*[3] | - | +/- | - | - | | - | NA | NA | + | - | - | NA | NA | - | - | NA | - | NA | - |
| **Mouse** | [Tabula Muris](https://tabula-muris.ds.czbiohub.org/) | NA | NA | +/- | - | | - | - | +/- | + | - | NA | NA | + | NA | - | NA | NA | - | NA |

GCGR, Glucagon receptor; NA, not available; +, expressed; -, not expressed; +/- , possibly expressed

1. Söllner JF, Leparc G, Hildebrandt T, Klein H, Thomas L, Stupka E, et al. An RNA-Seq atlas of gene expression in mouse and rat normal tissues. Sci Data. 2017;4:170185. Epub 20171212. doi: 10.1038/sdata.2017.185. PubMed PMID: 29231921; PubMed Central PMCID: PMCPMC5726313.

2. Roller M, Stamper E, Villar D, Izuogu O, Martin F, Redmond AM, et al. LINE retrotransposons characterize mammalian tissue-specific and evolutionarily dynamic regulatory regions. Genome Biol. 2021;22(1):62. Epub 20210218. doi: 10.1186/s13059-021-02260-y. PubMed PMID: 33602314; PubMed Central PMCID: PMCPMC7890895.

3. Naqvi S, Godfrey AK, Hughes JF, Goodheart ML, Mitchell RN, Page DC. Conservation, acquisition, and functional impact of sex-biased gene expression in mammals. Science. 2019;365(6450). doi: 10.1126/science.aaw7317. PubMed PMID: 31320509; PubMed Central PMCID: PMCPMC6896219.

4. Dunphy JL, Taylor RG, Fuller PJ. Tissue distribution of rat glucagon receptor and GLP-1 receptor gene expression. Mol Cell Endocrinol. 1998;141(1-2):179-86. doi: 10.1016/s0303-7207(98)00096-3. PubMed PMID: 9723898.

5. Hansen LH, Abrahamsen N, Nishimura E. Glucagon receptor mRNA distribution in rat tissues. Peptides. 1995;16(6):1163-6. doi: 10.1016/0196-9781(95)00078-x. PubMed PMID: 8532603.
