# Supplementary material for: Preclinical evaluation of a protracted GLP-1/glucagon receptor co-agonist: Translational difficulties and pitfalls
Source: PLoS One. 2022 Mar 4;17(3):e0264974. doi: 10.1371/journal.pone.0264974 (PMC8896685; doi:10.1371/journal.pone.0264974)
Supplement: S3 Table — (DOCX) [file pone.0264974.s006.docx]

| **Human** | **Study/Database** | **Brain** | **Adrenal** | **Colon** | **GI tract** | **Heart** | **Kidney** | **Liver** | **Muscle** | **Nerve** | **Pancreas** | **Salivary gland** | **Skin** | **Spleen** | **Testis** | **Thyroid** | **Parathyroid gland** |
| --- | --- | --- | --- | --- | --- | --- | --- | --- | --- | --- | --- | --- | --- | --- | --- | --- | --- |
| **GCGR** | https://www.gtexportal.org/home/gene/GCGR | - | - | +/- | - | - | + | + | - | + | - | - | +/- | - | +/- | - | NA |
| **GCGR** | <https://tabula-sapiens-portal.ds.czbiohub.org/home> | NA | NA | - | - | - | - | + | - | NA | - | - | - | - | NA | NA | NA |
| **GCGR** | https://www.proteinatlas.org/ENSG00000215644-GCGR | - | +/- | - | - | - | + | + | - | NA | +/- | - | - | - | - | - | +/- |
| **GLP-1R** | https://www.gtexportal.org/home/gene/GLP1R | - | - | - | - | +/- | - | - | - | - | + | - | - | - | - | - | NA |
| **GLP-1R** | <https://tabula-sapiens-portal.ds.czbiohub.org/home> | NA | NA | - | - | - | - | - | - | NA | + | + | - | - | NA | NA | NA |
| **GLP-1R** | https://www.proteinatlas.org/ENSG00000112164-GLP1R | + | - | - | +/- | + | - | - | - | NA | + | + | - | - | - | - | - |

**S3 Table: Tissue distribution of glucagon and GLP-1 receptors in humans**

Glucagon receptor, GCGR; NA, not available; +, expressed; -, not expressed; +/-, possibly expressed
