# Supplementary material for: Preclinical evaluation of a protracted GLP-1/glucagon receptor co-agonist: Translational difficulties and pitfalls
Source: PLoS One. 2022 Mar 4;17(3):e0264974. doi: 10.1371/journal.pone.0264974 (PMC8896685; doi:10.1371/journal.pone.0264974)
Supplement: S1 File — (DOCX) [file pone.0264974.s008.docx]

**S1 File. Compound synthesis**

All peptides were prepared by solid phase peptide synthesis using Fmoc based chemistry on a Prelude Solid Phase Peptide Synthesizer from Protein Technologies on a Rink Amide AM polystyrene resin (Novabiochem) or a ChemMatrix H-PAL resin. Introduction of the substituent on the epsilon-nitrogen of a lysine was achieved using a Lysine protected with Mtt (Fmoc-Lys(Mtt)-OH). The Mtt group was removed using HFIP/DCM/TIPS (75:20:5) (5 min). Cleavage of peptides from the resin were achieved using (TFA/TIPS/H2O (95:2.5:2.5 V/V) for 2.5h). The crude peptides were purified by reversed-phase preparative HPLC (Waters Delta Prep 4000) on a column comprising C18-silica gel. Elution was performed with an increasing gradient of MeCN in MQ water comprising 0.1% TFA. The fractions were then analysed by UPLC and LCMS and pure fractions were combined and freeze dried.
