# Supplementary material for: Preclinical evaluation of a protracted GLP-1/glucagon receptor co-agonist: Translational difficulties and pitfalls
Source: PLoS One. 2022 Mar 4;17(3):e0264974. doi: 10.1371/journal.pone.0264974 (PMC8896685; doi:10.1371/journal.pone.0264974)
Supplement: S2 File — (DOCX) [file pone.0264974.s009.docx]

**S2 File. Pharmacokinetics in mice and rats**

Lean rats received a single intravenous (i.v.) injection in the tail vein of a GLP-1-glucagon receptor co-agonist NN1151, NN1177 or NN1359 at a dose of 10 nmol/kg. At t=0, 5 min, 10 min, 30 min, 2 h, 4 h, 8 h, 12 h, 24 h, 32 h, 48 h, 56 h, and 72 h post dosing, plasma was sampled from the sublingual plexus for analysis of plasma exposure measured by LOCI.

Lean mice received a single subcutanous (s.c.) injection of the GLP-1 reference compound at a dose of 10 nmol/kg. At t=0, 5 min, 30 min, 1 h, 3 h, 6 h, 10 h, 24 h and 48 h post dosing, plasma was sampled from the sublingual plexus for analysis of plasma exposure measured by LOCI.

Plasma concentration-time profiles were analysed using non-compartmental analysis (NCA) using Phoenix WinNonlin Professional 6.3 (Pharsight, Mountain View, CA, US). Calculations were performed using full concentration-time profile values or sparse sampling concentration-time values as appropriate.

The terminal half-lives for NN1151, NN1177 and NN1359 were found to be 4 h, 8 h, and 11 h, respectively. The terminal half-life of the GLP-1 reference compound was found to be 11 h.
