# Supplementary material for: Preclinical evaluation of a protracted GLP-1/glucagon receptor co-agonist: Translational difficulties and pitfalls
Source: PLoS One. 2022 Mar 4;17(3):e0264974. doi: 10.1371/journal.pone.0264974 (PMC8896685; doi:10.1371/journal.pone.0264974)
Supplement: Attachment — Submitted filename: Response to Reviewers.docx [file pone.0264974.s010.docx]

**Dear Editor and Reviewers,**

Thank you for reviewing and considering our manuscript for publication in PLOS ONE. It is our belief that we with present letter and accompanying revised manuscript have addressed your requirements for publication.

1. We have ensured that headings, figures, references and tables are correctly formatted and named.
2. Thank you for pointing to the discrepancy between ‘funding information’ and ‘financial disclosure´. There was no external funding source for these studies. All studies were supported by Novo Nordisk A/S. The information has been corrected in the funding information.
3. Thank you for pointing to the lack of sponsor information. Please use this amended competing interest information:

All authors are or were full time employees at Novo Nordisk A/S and hold a minor share portion as part of their employment. Novo Nordisk A/S manufactures and markets pharmaceuticals related to diabetes, obesity and other chronic diseases and has intellectual property rights for several inventions related to these diseases. This does not alter our adherence to PLOS ONE policies on sharing data and materials.

4. Reviewer’s comments:

Reviewer #1:

We acknowledge the reviewer’s wish for including more information on antibodies used for compound exposure analysis. We have now added more details to the manuscript (Page 9, line 192-197)

Reviewer #2:

We agree with the reviewer that it would be interesting to compare body weight profiles of NN1177 with MEDI0382, as well as with other co-agonists that have made it into clinic. We do however feel this comparison is hard to make and conclusions will be somewhat speculative in the absence of head-to head data in similar *in vitro* assay systems. Based on the body weight profiles we can speculate on the receptor balance in competitor molecules, but we do not think such comparisons/speculations will contribute much to the increased understanding of our findings and is therefore not within the scope of the present manuscript.

We thank the reviewer for pointing out the lack of information relating to the choice of fatty acid linker. We have now elaborated on the reasoning, which is added to the manuscript (Page 5-6, lines 106-109) and have also included and additional reference (nr 22, Schlein *et al.* 2017) describing the technique used for physical stability measurements.

Yours Sincerely,

Charlotta Dornonville de la Cour
